# Childhood Characteristics of Adolescent Inpatients with Early-Onset and Adolescent-Onset Disruptive Behavior

**DOI:** 10.1007/s10862-012-9283-8

**Published:** 2012-03-30

**Authors:** Sjoukje B. B. de Boer, Floortje V. A. van Oort, Marianne C. H. Donker, Fop Verheij, Albert E. Boon

**Affiliations:** 1 De Fjord, Centre of Orthopsychiatry and Forensic Youth Psychiatry, Poortmolen 121, 2906 RN Capelle aan den IJssel, The Netherlands; 2 Erasmus MC-Sophia (Sophia Children’s Hospital), Erasmus MC University Medical Center, Rotterdam, The Netherlands; 3 Ministry of Health, Welfare and Sports, The Hague, The Netherlands; 4 De Jutters, Centre of Youth Mental Healthcare Haaglanden, The Hague, The Netherlands; 5 Erasmus MC University Medical Center, Rotterdam, The Netherlands

**Keywords:** Adolescents, Antisocial behavior, Psychiatric inpatients, Childhood characteristics

## Abstract

Childhood characteristics are associated with life-course-persistent antisocial behavior in epidemiological studies in general population samples. The present study examines this association in an inpatient sample. The purpose is to identify easily measurable childhood characteristics that may guide choice of treatment for adolescent psychiatric inpatients with severe disruptive behavior. Patients (*N* = 203) were divided into two groups with either early-onset (EO) or adolescent-onset (AO) disruptive behavior, based on ages at which professional care was used for disruptive behavior, referral to special education, and criminal offences. Both groups differed on several childhood characteristics. No gender differences in these characteristics were found. Logistic regression analysis indicated that individuals with grade retention in primary school, childhood impulsive behavior, and a history of physical abuse, had the highest probability of being member of the EO group. These characteristics are reasonably easy to identify, likely apply to other clinical samples as well, and may help clinicians to target their treatment.

## Introduction

In epidemiological research, Moffitt (1993) found that a relatively small proportion of the population engaged in antisocial behavior at a very young age. In about a quarter (Veenstra et al. 2009) to half (Moffitt et al. 1996) of these children this disruptive behavior was limited to childhood, the remainder of this group were labeled life-course-persistent (LCP), because of the continuous course of their antisocial behavior. A larger group was found to be involved in antisocial behavior only during adolescence, therefore their behavior was labeled adolescence-limited (AL). Although during adolescence these groups do not differ in frequency and seriousness of offending, it is argued that they differ in etiology, prognosis and classification of their behavior as either normative or pathological (Moffitt 1993, 2003; Moffitt et al. 2002, 2008).

This paper tests the classification of these two hypothetical prototypes in an inpatient sample of youths who had severe disruptive behavior combined with psychiatric disorders. Disruptive behavior includes aggression, oppositional behavior, status offences (e.g. running away, truancy, substance abuse), and property violations (e.g. lying or deceiving, selling drugs, vandalism). Replication of findings from general population studies in clinical populations is important, because findings then become clinically useful (Moffitt et al. 2008). In clinical populations it is often difficult to classify adolescents as having LCP or AL disruptive behavior, as information of childhood disruptive behavior is not always present, or difficult to collect retrospectively. As persistence of the behavior is even more difficult to determine retrospectively, and it is unclear whether disruptive behavior in adolescence will persist in later life we choose to use the terms early-onset (EO) and adolescent-onset (AO). Adolescents with severe disruptive behavior who also had disruptive behavior in childhood, we label as EO; adolescents in this group have a significant chance to further develop as those in Moffitt’s LCP group. The group of adolescents who did not have disruptive behaviors in childhood, we label as AO; adolescents in this group will presumably develop as those in Moffitt’s AL group.

A set of risk indicators that is relatively easy to collect, or that is already collected in clinical process, and is strongly predictive of EO versus AO disruptive behavior, helps the clinician to assess the likelihood that an adolescent belongs to the LCP or the AL group. This may be of importance for choices with regard to treatment. Moffitt argued that both groups need intervention, but that they require different intervention goals and approaches (Moffitt et al. 2008). The causes of LCP antisocial behavior may be completely different from the causes of AL antisocial behavior, but beyond that the personal, educational and social development of the LCP group may have been seriously hampered by the sheer duration of their problems. The main goal of this study is to identify factors that diagnosticians can use to differentiate between the subtypes LCP and AL disruptive behavior in a clinical setting. According to Moffitt (1993), the strongest predictors of LCP antisocial behavior were individual and family characteristics. It is expected that this will also be so for EO disruptive behavior. Individual factors include under-controlled temperament (Aguilar et al. 2000; Moffitt 2003; Moffitt and Caspi 2001; Moffitt et al. 1994), neurological abnormalities and delayed motor development (Moffitt 1993), low intellectual ability (Fergusson et al. 2000; Moffitt 2003; Moffitt and Caspi 2001; Moffitt et al. 1994; Nagin and Farrington 1992; Odgers et al. 2008), reading difficulties (Moffitt 2003; Moffitt and Caspi 2001; Moffitt et al. 1994; Odgers et al. 2008), low school achievement (Chung et al. 2002; Moffitt 1993; Vaughn et al. 2011), poor scores on neuropsychological tests, caused by birth complications for example (Moffitt 2003; Moffitt and Caspi 2001; Moffitt et al. 1994; Tibbetts and Piquero 1999), hyperactivity and/or attention problems (Fergusson et al. 2000; Moffitt and Caspi 2001; Moffitt et al. 1994; Nagin and Tremblay 2001; Odgers et al. 2008; Wiesner and Capaldi 2003), low heart rate (Moffitt 2003; Moffitt and Caspi 2001; Moffitt et al. 1994; Odgers et al. 2008), psychopathic personality traits, violent behavior (Moffitt and Caspi 2001; Moffitt et al. 2002, 1994), and broad psychiatric comorbidity (Vaughn et al. 2011).

Family and context factors associated with LCP antisocial behavior include: having a teenage single parent (Fergusson et al. 2000; Moffitt and Caspi 2001; Nagin and Tremblay 2001), having a single parent at birth (Aguilar et al. 2000; Fergusson et al. 2000; Kjelsberg 1999; Tibbetts and Piquero 1999), maltreatment (mothers who were harsh or neglectful, harsh or inconsistent discipline, physical abuse, sexual abuse, child abuse) (Aguilar et al. 2000; Dean et al. 1996; Moffitt and Caspi 2001; Odgers et al. 2008; Patterson et al. 1998; Wiesner and Capaldi 2003), much family conflict (Fergusson et al. 2000; Moffitt and Caspi 2001; Odgers et al. 2008), inadequate parenting (low parental supervision, inconsistent discipline)(Chung et al. 2002; Moffitt and Caspi 2001; Moffitt et al. 2002; Nagin and Farrington 1992; Odgers et al. 2008; Patterson et al. 1998; Wiesner and Capaldi 2003), many changes of primary care taker (Kjelsberg 1999; Moffitt and Caspi 2001; Nagin and Farrington 1992; Patterson et al. 1998; Tibbetts and Piquero 1999), and sibling deviance (Moffitt 1993).

Parental characteristics associated with LCP antisocial behavior were: mothers with poor mental health (Moffitt and Caspi 2001; Odgers et al. 2008; Shaw et al. 1996), parental criminal conviction (Fergusson et al. 2000; Kjelsberg 1999; associated with AL: Nagin and Farrington 1992; Odgers et al. 2008), parental deviance (Moffitt 1993), parental alcoholism, alcohol problems or illicit drug use (Fergusson et al. 2000), low educational attainment or IQ of the mother (Fergusson et al. 2000; Nagin and Tremblay 2001; Odgers et al. 2008), and low family socio-economic status (SES) (Fergusson et al. 2000; Fontaine et al. 2009; Kjelsberg 1999; Moffitt and Caspi 2001; Odgers et al. 2008; Patterson et al. 1998; Tibbetts and Piquero 1999).

Individuals on the AL path tended to have backgrounds that were normative (Moffitt and Caspi 2001). AL offending was assumed to be most strongly related to associations with deviant peers. Attitudes toward adulthood and autonomy, cultural and historical context and age were considered the strongest predictors of short-term offending (Moffitt 1993).

Thus far, the distinction in EO and AO antisocial behavior has hardly been made in clinical practice. We expect that both groups are represented in our sample and that factors that were found to associate with LCP antisocial behavior in epidemiological studies are also associated with EO disruptive behavior in a clinical sample. The purpose of the present study is to find individual, parental, and family and context risk factors that were present in childhood, that are relatively easy to collect, and are strongly predictive of EO versus AO disruptive behavior in a clinical sample of adolescents with severe disruptive behavior.

## Method

### Setting

The present study was conducted at De Fjord, an orthopsychiatric and forensic youth treatment facility in Rotterdam, The Netherlands. In The Netherlands, the term orthopsychiatry entails specialized treatment of youngsters diagnosed with severe disruptive behavior (that may or may not include offending) in combination with one or more psychiatric disorders. The Fjord offers outpatient and day treatment, and a specialized residential treatment program. Patients are eligible for treatment if they are referred by other specialized youth care institutions, i.e. institutions that are predominantly focused on developmental, psychiatric or criminal problems in children and adolescents. In addition to referral, patients must meet the following inclusion criteria: age between 16 and 20 years, presence of severe behavioral as well as psychiatric problems, and (a history of) previous treatment. These criteria result in a patient sample with severe and complex problems that were not resolved by treatments elsewhere. Patients functioning below borderline intellectual level (IQ <70), with predominant addiction problems, or with severe recidivist criminal conduct for which specialized, individual forensic treatment is indicated, are not eligible for treatment.

### Procedure

All patients admitted between 1995 and 2008 were included in the study. After a verbal description of the study to the subjects, written informed consent was obtained. All patients (*N* = 223) agreed to participate. When patients were under age 16, in accordance with the statutory requirement in the Netherlands, informed consent was also obtained from the parents. The statistical analyses in present study were performed for 203 patients for whom the age of onset was determined (91.0 % of the sample).

Disruptive behavior during childhood, its age of onset, and other childhood characteristics were obtained by using multi-informant (adolescent, parent and therapist), multi-method (self-report, interview, records from mental health care institutions where patients had previously been treated) information. The adolescent was interviewed by the researcher, and the therapist reported all known characteristics of the youngster via a questionnaire. This information was partly based on information reported by parents and/or referring professional (e.g. guardian or probation officer) during the intake procedure. Information from records was obtained by the researcher. A behavior or characteristic was considered present when mentioned by at least one of the sources, and absent when not present according to all sources. When information was not available, it was coded unclear (or missing, depending on the reason for unavailability). Some characteristics were considered too aggravating to be asked directly by the researcher (e.g. sexual abuse, physical abuse), and were therefore obtained from the therapist (i.e. via the therapist who asked the adolescent) and from records.

### Measures

*Presence of disruptive behavior during childhood* was determined, based on the age at which help was sought because of disruptive behavior, special education was indicated due to disruptive behavior, and the age at which the youngster started to commit criminal offences. For each individual the presence and age of onset of disruptive behaviors was determined. For age of onset the earliest age reported by any of the sources was used. Disruptive behavior included aggression (*overt, destructive*: e.g. physical abuse, sexual offences, threatening someone), oppositional behavior (*overt, non-destructive*: e.g. disobedient, doing things own way), status offences (*covert, non-destructive*: e.g. running away, truancy, substance abuse), and property violations (*covert, destructive*: e.g. lying or deceiving, selling drugs, vandalism) (Frick et al. 1993). Subsequently, a distinction was made in two groups labeled early-onset (EO) and adolescent-onset (AO). The EO group will most likely develop as a LCP group and the AO group as an AL group. Patients with disruptive behavior starting prior to age 12 were considered members of the EO group and those whose disruptive behavior started from age 12 on were members of the AO group (De Boer et al. 2007, 2011). In the sample, both EO (*n* = 134, 66 %) and AO (*n* = 69, 34 %) groups were found.

To describe the sample, information on current DSM diagnoses, type of referral, prior experience with institutionalized care, and penal and civil measures was collected. Penal measures comprised: “*probation*”, “*mandatory treatment order*”, and “*conditional mandatory treatment order*”. Civil measures comprised “*supervision order*”, and *“involuntary commitment”*.

The childhood risk indicators were grouped into four categories: individual, family and context, parental, and system characteristics.

### Individual Child Characteristics

Information on gender, IQ (70,9 % had an IQ score measured by former institutions - WAIS: 48.6 %; WISC-R: 18.5 %; Raven: 6.9 %; or other measures: 36.0 %), school achievement (grade retention in primary school, and age at grade retention), and the presence of impulsive behavior and/or concentration problems was collected. The latter was considered present when professional help was sought because of this behavior.

### Family and Context Characteristics

Information on single parenthood at birth, parent’s divorce (birth—age 11), the number of changes in caregiver or changes of home environment, and maltreatment was collected. Maltreatment was divided into whether or not patients had been either physically or sexually abused.

### Parental Characteristics

Information on mental health care received by at least one parent, and parental conviction(s) for crimes was collected. Parents occupational level was coded into five categories (no occupation, housekeeper, without work or unfit for work; occupation without qualification; low vocational occupation; intermediate vocational occupation; high vocational or academic occupation) and subsequently, the highest level of occupation of the parents was determined, indicating social economic status (SES). Next to occupational level we asked for the mother’s employment status.

### System Characteristics

System characteristics included placement outside of the home before age 12 (yes/no). Also, when relevant, the age at court custody (placement outside of the home) was recorded.

## Statistical Analysis

All analyses were performed using the Statistical Package for the Social Sciences, version 17.0 (SPSS 2008). As all of the characteristics were risk indicators for onset of disruptive behavior at a young age, it was expected that they would be more present in the EO group than in the AO group, and we tested one-sided for differences. Although many childhood factors that were associated with LCP antisocial behavior seem to apply to females as well, there are indications that gender differences exist (e.g. Barnes and Beaver 2010; Eme 2007; Odgers et al. 2008). For this reason we checked for gender specificity of the characteristics. First, with chi-square tests (categorical variables) or student *t*-tests (continuous variables), the EO group was compared with the AO group. A level of significance of *p* < .003 (Bonferroni correction) was chosen to account for the number of characteristics tested. Second, in a logistic regression analysis (dependent EO vs AO), all characteristics were included that differed significantly (*p* < .05) between EO and AO groups as independent variables, as well as sex. We tested for sex specific characteristics by including interaction terms with sex. Interactions with a *p*-level of <.10 were included in the model. The Nagelkerke *R*-square of the model was used as measure for effect size.

## Results

Table 1 describes the patients included in the study. The sample was comprised of 48 female and 155 male patients with a mean age of 17.7 years. Although the level of intelligence (mostly measured at the institutions that requested the admission) of the sample was approximately average, the educational attainment was relatively low. Over 20 % of the sample had a penal measure and approximately 50 % had a civil measure. Some of these individuals had a civil measure and a penal measure. In addition to their psychiatric problems, all patients in the sample displayed severe disruptive behavior.

**Table 1:** Characteristics of the sample

*N* = 203	*N* ^a^	*N* mean	(%) (SD)
Characteristic			
Sex (male)	203	155	(76.4 %)
Age (years)	203	17.7	(1.2)
Ethnicity Dutch	203	148	(72.9 %)
Educational level	198		
Not attending school		32	(16.2 %)
Special education		18	(9.1 %)
Pre-vocational or junior general secondary education		125	(63.1 %)
Senior general secondary or pre-university education		23	(11.6 %)
Referral	203		
Youth care		92	(45.3 %)
Youth mental health care		76	(37.4 %)
Judicial institutions		35	(17.2 %)
Penal measure	203	43	(21.2 %)
Civil measure	203	102	(50.2 %)
Penal and civil measure	203	11	(5.4 %)
Number of DSM diagnoses (Axis I)	197	2.7	(1.2)
DSM diagnoses (Axis I)	197		
Conduct disorder		79	(40.1 %)
Oppositional defiant disorder		55	(27.9 %)
Schizophrenia and related disorders		45	(22.8 %)
Mood disorder		34	(17.3 %)
Autism spectrum disorder		33	(16.8 %)
Attention-deficit/hyperactivity disorder (ADHD)		31	(15.7 %)
Anxiety disorder		21	(10.7 %)
Personality disorders (NOS and cluster B)	198		
Diagnosed		57	(28.8 %)
Suspected		102	(51.5 %)
Institutionalized care (prior to De Fjord)	202		
Yes		197	(97.5 %)
Former admissions		2.9	(2.0)
Onset of disruptive behavior	203		
Early-onset (<12 years)		134	(66.0 %)
Adolescent-onset (>11 years)		69	(34.0 %)

^a^N Number of patients for whom information about the characteristic was available

Table 2 shows the characteristics by EO and AO classification. Males were overrepresented in the EO group (82 % vs 18 % females) and in the AO group (65 % vs 35 % females). Table 2 shows that EO and AO groups differed on individual characteristics (in occurrence and age at grade retention in primary school, and in impulsive behavior), family and context characteristics (parental divorce (before child age 11), the number of changes in home environment, and physical abuse), parental characteristics (employment of the mother), and system characteristics (age at first placement outside of the home). The EO and AO groups did not differ on IQ, single parent at birth, sexual abuse, mental health care received by at least one of the parents, parental conviction, or SES.

**Table 2:** Childhood characteristics by onset of disruptive behaviors and by gender (*n* = 203)

		EO ♂ (*n* = 110)	AO ♂ (*n* = 45)	EO ♀ (*n* = 24)	AO ♀ (*n* = 24)	EO versus AO
	N^a^	*n* (%)/M (SD)	*n* (%)/M (SD)	*n* (%)/M (SD)	*n* (%)/M (SD)	*p*-value
Individual						
Intellectual ability (IQ)	135	99.16 (12.97)	97.94 (12.51)	98.17 (11.93)	98.36 (15.13)	0.344
Grade retention in primary school	146	20 (26.7 %)	4 (12.1 %)	5 (27.8 %)	2 (10.0 %)	0.023*
Age at grade retention	72	8.85 (3.83)	10.80 (2.65)	8.67 (3.80)	10.33 (15.13)	0.004*
Impulsive behavior	177	57 (59.4 %)	6 (14.3 %)	9 (42.9 %)	1 (5.6 %)	0.000**
Family and context						
Single parent at birth	195	9 (8.4 %)	2 (5.0 %)	4 (16.7 %)	4 (16.7 %)	0.500
Parents divorced (birth—age 11)	202	54 (49.5 %)	12 (26.7 %)	16 (66.7 %)	10 (41.7 %)	0.004*
Number of changes in home environment	201	3.58 (2.52)	2.49 (1.92)	4.46 (2.83)	3.83 (2.94)	0.022*
Physical abuse	178	47 (47.5 %)	10 (25.6 %)	11 (55.0 %)	7 (35.0 %)	0.009*
Sexual abuse	164	10 (11.1 %)	6 (15.8 %)	12 (63.2 %)	11 (64.7 %)	0.092
Parental						
Mental healthcare parents	124	35 (52.2 %)	10 (31.3 %)	5 (45.5 %)	8 (57.1 %)	0.131
Conviction parents	124	7 (11.5 %)	1 (3.2 %)	7 (41.2 %)	2 (13.3 %)	0.065
Highest occupation of both parents	177	–	–	–	–	0.190
No		8 (8.2 %)	2 (11.8 %)	5 (12.2 %)	4 (18.2 %)	
Without qualification		6 (6.2 %)	0 (0 %)	3 (7.3 %)	4 (18.2 %)	
Low qualification		26 (26.8 %)	6 (35.3 %)	9 (22.0 %)	5 (22.7 %)	
Intermediate qualification		40 (41.2 %)	8 (47.1 %)	19 (46.3 %)	7 (31.8 %)	
High qualification		17 (17.5 %)	1 (5.1 %)	15 (12.2 %)	2 (9.1 %)	
Working mother	174	71 (74.0 %)	22 (55.0 %)	10 (62.5 %)	11 (50.0 %)	0.009*
System						
Placement outside of the home < age 12	202	15 (13.6 %)	2 (4.5 %)	6 (25.0 %)	4 (16.7 %)	0.129
Age out placement outside of the home < age 12	27	5.60 (3.18)	9.50 (0.71)	4.50 (2.26)	7.50 (3.11)	0.020*

*EO* early-onset; *AO* adolescent-onset

^a^N Number of patients for whom information about the characteristic was available

*p < .05 (one-tailed)

**p < .003 (one-tailed), significant after Bonferroni correction

### Sex

When subsequently males and females of the EO group were compared, two differences in childhood characteristics were found: compared with EO males significantly more EO females had a parent who had been convicted of a crime (41 % versus 12 %, *p* = .014) and significantly (*p* < .0001) more females (63 %) had been sexually abused compared with males (11 %). This was also found for the AO group (65 % females, 16 % males, *p* = .001).

### Logistic Regression

The significant characteristics of Table 2 were entered into a logistic regression equation. The logistic regression analysis was performed to test the predictive value of the variables on (the dichotomous dependent variable) EO disruptive behavior. No significant differences between males and females were found after testing for interaction effects.

The first model shows the bivariate odds ratios. Each of the characteristics significantly predicted membership of the EO group, except for age at placement outside of the home. Odds ratios ranged from 1.14 (number of changes in the home environment) to 9.80 (impulsive behavior), with effect sizes ranging from 0.03 to 0.26. The multivariate model showed three significant independent predictors of EO-membership: grade retention, impulsive behavior and physical abuse (Table 3). The model was statistically significant (*X* ^*2*^ (7, N = 98) = 29.72, *p* < .0001), indicating that the model was able to distinguish the patients with EO from those with AO disruptive behavior. The model as a whole explained 36 % (Nagelkerke R square) of the variance in onset, and correctly identified 75.5 % of cases.

**Table 3:** Logistic regression analysis of the associations between childhood characteristics and EO and AO disruptive behavior

	Model 1			Model 2		
	N	OR	95 % CI	R2	OR	95 % CI
Sex	203	2.44	(1.26–4.75)*	0.05	1.43	(0.39–5.32)
Grade retention in primary school	146	2.88	(1.10–7.56)*	0.05	4.18	(1.12–15.68)*
Age at grade retention^a^	72	0.82	(0.69–0.97)*	0.12		
Impulsive behavior	177	9.80	(4.11–23.36)**	0.26	6.01	(1.91–18.91)**
Parents divorced	203	2.41	(1.31–4.43)*	0.06	1.49	(0.52–4.30)
Number of changes in the home environment	201	1.14	(1.00–1.29)*	0.03	1.01	(0.80–1.29)
Physical abuse	178	2.35	(1.20–4.58)*	0.05	3.64	(1.09–12.18)*
Working mother	174	2.30	(1.20–4.39)*	0.05	2.29	(0.76–6.88)
Age placement outside of the home < age 12^a^	27	0.68	(0.45–1.01)	0.25		

Model 1: univariate; model 2: multivariate. Multivariate model: *n* = 98; *EO* early-onset; *AO* adolescent-onset; *OR* odds ratio; 95 % CI: 95 % confidence interval; R2: Nagelkerke R2

^a^Due to small n not included in the multivariate model.

*p < .05 ** *p* < .003, significant after Bonferroni correction. Nagelkerke R2 model 2: 0.36

## Discussion

In previous research, in our clinical sample of inpatient adolescents with disruptive behavior and psychiatric disorders, we were able to make the distinction in EO and AO based on retrospective data (De Boer et al. 2007, 2011). The main goal of this paper was to identify factors that diagnosticians can use to differentiate between the subtypes EO and AO disruptive behavior in a clinical setting. This was done to help clinicians identify characteristics relevant to the choice of treatment for each group. Because of this practical purpose, we looked for characteristics that may easily be available in routine clinical practice.

As expected, the EO group showed higher levels of risk in childhood, compared to the AO group, including characteristics indicating inherited or acquired neuropsychological deficits and environmental risk factors (i.e. mean age at grade retention, grade retention in primary school, and prevalence of impulsive behavior). Besides, the EO and AO groups differed significantly on many of the other childhood risk factors (the number of changes in home environment, parental divorce (before age 11 years), physical abuse, employment of the mother, and mean age at placement outside of the home). Logistic regression yielded grade retention in primary school, impulsive behavior and physical abuse to be significantly correlated to EO disruptive behavior.

Differences in IQ were not found, but youngsters with very low cognitive ability were not included in this study because they were not eligible for treatment at De Fjord. Furthermore, the EO and AO groups did not differ on single parent at birth, sexual abuse, mental health care received by at least one of the parents, parental conviction, or SES.

It is important to note that early onset (and probably life course persistence) of disruptive behavior does occur in females. Females with EO disruptive behavior resembled their male counterparts to a great extend, they only differed on two characteristics. Compared with males of the EO group, more females with EO disruptive behavior had a parent who had been convicted of a crime. Sexual abuse was much more prevalent in females than in males, but this was found for both EO and AO groups, indicating that it was not related to the age onset of disruptive behavior. When tested for interaction effects, no significant sex differences were found. The number of girls in our sample, and the selection of characteristics were limited, but our findings do not support gender differences in these characteristics in their value for signaling EO disruptive behavior in adolescents. Gender differences may be present in biological or neurodevelopmental factors involved in the development of EO disruptive behavior (Eme 2007, 2009; Kjelsberg 1999).

It has to be noted that, methodologically, our set of variables did not permit an exhaustive test of all childhood variables that have been pinpointed to be involved in the development of LCP and AL antisocial behavior (e.g. peer characteristics, biological influences or neurodevelopmental factors). Also, the variables were not gathered at fixed moments during the early life of the patients as in epidemiological studies, but obtained retrospectively after admission. Some variables may have varied over time (e.g. child abuse, mental health of parents), but we presume that they have been considerably stable. Finally, comparison of our retrospective findings with epidemiological findings must be made with caution, because some retrospective measures (e.g. psychosocial variables) have low levels of agreement with prospective measures (Henry et al. 1994). In the present study, this was partly intercepted by using multi-informant information.

Many of the factors of epidemiological research that were found to be associated with EO disruptive behavior were also found to be associated with EO disruptive behavior in a highly selective clinical sample with severe disruptive behavior and co-occurring psychiatric disorders. This suggests that the factors associated with EO disruptive behavior probably also apply to other clinical (and non-clinical) samples with less severe psychiatric disorders and disruptive behavior. More research should be conducted among clinical and non-clinical samples to confirm this generalization.

In our study, we found characteristics that distinguish patients with EO from those with AO. We identified three independent childhood characteristics that predicted membership of the early onset group: grade retention in primary school, impulsive behavior and being physically abused. Other characteristics partly overlap in their ability to identify EO disruptive behavior. As these characteristics are not systematically collected in clinical practice, clinicians should attempt to collect information on as many as possible. The chance that the adolescent has EO disruptive behavior strongly increases in the presence of one or more of these characteristics. Because the characteristics are reasonably easy to identify, they may help clinicians to target their treatment. Adolescents with EO disruptive behavior probably benefit from interventions aimed at personality traits (psychopathic traits, impulsivity, hostility, alienation, and callousness), developing social skills (unless the individual has callous, unemotional psychopathic personality traits), aggression regulation, and education. Whereas adolescents with AO disruptive behavior probably benefit from interventions that prevent truancy or dropout from school, assertiveness therapy, or interventions that help to prevent (further) delinquent behavior.

In conclusion, in routine clinical practice information should be collected on early impulsive behavior, grade retention in primary school, and physical abuse, as this background of adolescents with severe disruptive behavior can help distinguish adolescents with early onset from those with adolescent onset disruptive behavior.
